# P097 Beyond sicca: cryoglobulinaemic vasculitis and multiorgan failure

**DOI:** 10.1093/rap/rkag113.127

**Published:** 2026-11-03

**Authors:** Sabine Bellammy, Sung-Hee Kim, Jasroop Chana

**Affiliations:** Buckinghamshire Healthcare NHS Trust, Stoke Mandeville, United Kingdom; Buckinghamshire Healthcare NHS Trust, Stoke Mandeville, United Kingdom; Buckinghamshire Healthcare NHS Trust, Stoke Mandeville, United Kingdom

## Abstract

**Introduction:**

A case of a patient who had a long history of Sjögren’s syndrome with main features of sicca symptoms, leukocytoclastic vasculitis and marginal zone lymphoma under surveillance. The patient presented acutely with respiratory distress and renal dysfunction and was subsequently diagnosed with cryoglobulinaemic vasculitis. This case showcases that although Sjögren’s syndrome is often seen as a benign disease, it can have severe sequelae that all rheumatologists need to be aware of.

**Case description:**

We present a 66 year old patient with a 16 year history of Sjögren’s syndrome and leukocytoclastic vasculitis (Ro52 and anti-centromere antibodies positivity). She was managed with hydroxychloroquine and occasional steroids. Her other medical problems include proteinuria and hypertension.

Prior to her acute admission, she was recently diagnosed with low grade B cell lymphoma after an incidental finding of lymphadenopathy on CT scan which was arranged to investigate dyspnoea. This was proven via histology and due to its indolent nature, it was managed through active surveillance.

While being under investigation, she developed new ascites, persistent proteinuria and worsening renal function. Renal and liver biopsies were subsequently arranged.

Unfortunately, whilst awaiting results, the patient presented to hospital acutely with fatigue, chest pain and shortness of breath. On examination, she required 2L oxygen and auscultation of the chest revealed bi-basal crackles and bilateral pitting oedema. JVP was not elevated. ECG was initially atrial fibrillation with fast ventricular rate, then reverted to sinus rhythm. Blood tests showed a raised CRP at 101 with normal white cell count, BNP was raised at 10000. Chest Xray showed bilateral infiltrates. She was initially treated for heart failure and pneumonia with diuretics and antibiotics.

Unfortunately, she further deteriorated and developed type 1 respiratory failure, CTPA showed ground glass changes bilaterally. ECHO showed ejection fraction of 55% and diastolic dysfunction. She was transferred to intensive care, antibiotics continued and pulsed with methylprednisolone. Renal function continued to decline requiring filtration as well as worsening respiratory function requiring intubation and ventilation. Renal biopsy results then became available showing cryoglobulinaemic vasculitis, discussed with renal and transferred for plasma exchange and rituximab.

Respiratory and renal function subsequently improved and patient was discharged on weaning course of prednisolone. She was further discussed by the haematology team to proceed with lymphoma dose rituximab.

**Discussion:**

This patient had stable Sjögren’s syndrome for many years with symptoms managed with hydroxychloroquine. She presented with acute respiratory distress and fluid overload. She deteriorated rapidly despite standard treatment with antibiotics and diuretics requiring ITU support. ITU team were contemplating withdrawing treatment as she continued to decline.

However, when she was treated for cryoglobulinaemic vasculitis with IVIG and rituximab, she made a dramatic improvement and was stepped down to the ward in a few days. This reminds us that although patients can get extremely unwell with acute presentation of systemic vasculitis, with prompt management, they can make a good recovery and rheumatologists must advocate for these patients.

What remains unexplained is the pulmonary involvement of her disease. Lung involvement in cryoglobulinaemic vasculitis is rare and more commonly presents as pulmonary haemorrhage rather than inflammatory changes. She also did not report haemoptysis nor she had a drop in haemoglobin. However, her lung disease was very responsive to steroids and immunosuppression therapy and there is no clear alternative diagnosis.

Additionally, this patient had a CT PET scan prior to admission which showed stable disease in view of her lymphoma. However, the acute deterioration and the new diagnosis of cryoglobulinaemic vasculitis raises possibility of transformation of higher-grade lymphoma. Recent studies suggest that patients with cryoglobulinaemic vasculitis has a six-fold increased risk of lymphoma. This is the rationale behind treatment with rituximab at lymphoma dose.

Finally, this patient had Sjögren’s for many years, preceding her diagnosis of Sjögren’s she also had leukocytoclastic vasculitis and shortly following the Sjögren’s diagnosis was found to have proteinuria. This may suggest that she was at risk of developing more severe disease.

**Key learning points:**

• The most common pulmonary involvement in Sjögren’s syndrome is interstitial lung disease. However other manifestations including lymphocytic interstitial pneumonitis and organising pneumonitis have been described. This patient presented acutely with worsening shortness of breath. CT imaging of her chest showed new widespread ground glass changes. This patient was positive for Ro52 and negative for Ro60 which has been associated with a higher risk of interstitial lung disease. This calls for early recognition and prompt treatment with immune suppression.

• Cryoglobulinaemic vasculitis can rarely be associated with pulmonary involvement including dyspnoea, pleuritic chest pain, pulmonary infiltrates and pulmonary haemorrhage. We are still unclear on the aetiology of her pulmonary disease. From the conference we would like to discuss the pulmonary involvement and the likely aetiology of this and whether investigations such as bronchoscopy may have helped in the diagnosis.

• Lymphoma is associated with Sjögren’s syndrome and patients should be asked about B symptoms routinely in clinic. This patient had Raynaud’s phenomena, vasculitis, rheumatoid factor positivity, hypocomplementemia and cryoglobulins which are associated with lymphoma development. If a patient develops cryoglobulinaemic vasculitis in context of low grade lymphoma, one should be looking out for possible transformation of lymphoma. Cryoglobulinemia is also associated with higher risk of extra glandular manifestations of disease as well. In this case the patient had unfortunately deteriorated before the diagnosis of cryoglobulinaemic vasculitis was known but once the biopsy result was available this was able to direct treatment.

• Sjögren’s syndrome is often thought to be a relatively benign disease causing sicca symptoms but it can have catastrophic multi-organ involvement. There are certain clinical and serological features that can help predict extra glandular disease and risk of lymphoma development. With prompt recognition and treatment, good recovery is possible.

